# Combining systems biology models of apoptosis provides superior predictions of the responsiveness of melanoma cells to cell death inducing drugs

**DOI:** 10.1186/1753-6561-9-S7-A10

**Published:** 2015-10-27

**Authors:** Paul Curtayne, Maximilian Wuerstle, Andreas Lindner, Jochen Prehn, Markus Rehm

**Affiliations:** 1 Royal College of Surgeons in Ireland, Dublin, Ireland; 2 Centre for Systems Medicine, Dept of Physiology and Medical Physics, Royal College of Surgeons in Ireland. Dublin, Ireland

## Background

Key to the clinical management of melanoma is the development of new diagnostic tools that predict individual patient prognosis and select from potential treatments those which may be effective. Identifying individual biomarkers in tumour cells to predict susceptibility to apoptotic cell death has thus far been largely unsuccessful, as apoptosis pathways show a high degree of signalling redundancy.

## Methods

DR_MOMP [1] and ApoptoCell [2] are mathematical systems biology models of the mitochondrial outer membrane permeabilisation and execution stages of the apoptosis pathway, respectively, that take into account the complex nature of apoptosis regulation. Both models use a network of ordinary differential equations representing measured protein concentrations and reaction kinetics. In this study we combine these models and compare model predictions to experimental measurements of cell death in a range of melanoma cell-lines that were treated with different cytotoxic agents.

## Results

The combined approach is found to outperform either individual model in predicting strong and weak responses to treatment with cell death inducing drugs.

## Conclusions

This work may provide a basis for the development of improved prediction tools for clinical treatment outcomes and treatment selection in melanoma.
